# Insulin-like growth factor-I peptides act centrally to decrease depression-like behavior of mice treated intraperitoneally with lipopolysaccharide

**DOI:** 10.1186/1742-2094-8-179

**Published:** 2011-12-21

**Authors:** Sook-Eun Park, Marcus Lawson, Robert Dantzer, Keith W Kelley, Robert H McCusker

**Affiliations:** 1 Integrated Immunology and Behavior Program, University of Illinois at Urbana-Champaign, Urbana, Illinois 61801-3873, USA; 2 Neuroscience Program, University of Illinois at Urbana-Champaign, Urbana, Illinois 61801-3873, USA; 3 Department of Animal Sciences, University of Illinois at Urbana-Champaign, Urbana, Illinois 61801-3873, USA; 4 Department of Pathology, University of Illinois at Urbana-Champaign, Urbana, Illinois 61801-3873, USA

**Keywords:** IGF-I, depression-like behavior, sickness, lipopolysaccharide

## Abstract

Centrally administered insulin-like growth factor (IGF)-I has anti-depressant activity in several rodent models, including lipopolysaccharide (LPS)-induced depression. In this study we tested the ability of IGF-I and GPE (the N-terminal tri-peptide derived from IGF-I) to alter depression-like behavior induced by intraperitoneal (i.p.) administration of LPS in a preventive and curative manner. In the first case, IGF-I (1 μg) or GPE (5 μg) was administered i.c.v. to CD-1 mice followed 30 min later by 330 μg/kg body weight i.p. LPS. In the second case, 830 μg/kg body weight LPS was given 24 h prior to either IGF-I or GPE. When administered i.p., LPS induced full-blown sickness assessed as a loss of body weight, decrease in food intake and sickness behavior. None of these indices were affected by IGF-I or GPE. LPS also induced depression-like behavior; assessed as an increased duration of immobility in the tail suspension and forced swim tests. When administered before or after LPS, IGF-I and GPE abrogated the LPS response; attenuating induction of depression-like behaviors and blocking preexistent depression-like behaviors. Similar to previous work with IGF-I, GPE decreased brain expression of cytokines in response to LPS although unlike IGF-I, GPE did not induce the expression of brain-derived neurotrophic factor (BDNF). LPS induced expression of tryptophan dioxygenases, IDO1, IDO2 and TDO2, but expression of these enzymes was not altered by GPE. Thus, both IGF-I and GPE elicit specific improvement in depression-like behavior independent of sickness, an action that could be due to their anti-inflammatory properties.

## Background

There is accumulating evidence that depression may develop in response to activation of the innate immune system [1-3]. Exposure of volunteers to a low dose of endotoxin induces depressed mood that correlated with cytokine expression, independent of sickness behaviors [4]. Recently, a low dose of endotoxin given to volunteers was for the first time shown to induce anhedonia, one of the primary features (diagnostic = DSM IV) for depression [5]. An increase in negative affect follows typhoid vaccine injections and similar to endotoxin exposure, these mood changes correlate with the induction of cytokine secretion [6]. Studies such as these provide a correlation between mood changes and inflammation, but a direct cause-effect link between activation of the innate immune system and mood changes came with human cytokine immunotherapy. Cancer immunotherapy and cytokine treatment for hepatitis C viral infection induces symptoms of depression in a significant percentage of patients [7,8]. These symptoms develop on a background of neurovegetative symptoms that are very similar to inflammation-induced sickness behavior [3]. Together with the Reichenberg study [4] showing a dissociation between depression and overt sickness, there is now strong evidence that depression does not fully overlap with sickness and that depression may be caused by cytokines in the brain.

In a rodent preclinical model, activation of the immune system reliably induces depression-like behavior assessed by several criteria including decreased preference for a sweetened (saccharin) solution over water, as an index of anhedonia, decreased sexual behavior [9], decreased preference for a sweetened (sucrose) solution over water, increased time of immobility during the forced swim test (FST) [10] and increased time of immobility during the tail suspension test (TST) [11]. LPS induces transient sickness with the changes in preference for a sweetened solution or immobility in the FST and TST still being evident after the disappearance of sickness; i.e. after locomotor activity, social exploration of a novel juvenile, body weight or food intake have normalized. These depression-like behaviors are reversed by anti-depressants and importantly by minocycline which attenuates LPS-induced expression of brain cytokines [9,11-14]. In all of these studies, depression-like behaviors continued after the acute immune response that was induced by LPS and the minocycline study clearly indicated that the cytokine response was requisite for the development of depression-like behaviors. These types of studies support the human data that inflammation is causative in the development or maintenance of depressive disorders.

Until recently, IGF-I has not been evaluated for anti-depression actions on a background of inflammation. We showed that i.c.v. IGF-I did not affect the acute sickness response that was induced by i.c.v. LPS. In contrast, IGF-I tempered cytokine expression and depression-like behavior [11]. In that study, IGF-I also increased the central expression of BDNF, a neurotrophin with well-characterized anti-depressant activity. For that work, gene expression was quantified in cDNA prepared from the entire perfused brain of mice [11,13]. Whether, the LPS or IGF-I responses were global or localized with a specific brain region was not examined. However, following a single LPS injection, pro-inflammatory cytokines, IL-1β, TNFα and IL-6 are all similarly elevated in the hippocampus and frontal cortex of mice [15]. Following repeated LPS injections, IL-1β is elevated in the frontal cortex, hippocampus and striatum [16]. These studies suggest that LPS induces a global inflammatory response within the brain and justified our previous use of total brain mRNA as the source for cDNA to quantify an immune response following LPS. However, it is clear from studies with humans that the frontal cortex plays a unique role in depression [17-20]. Similarly with rodents, electrical stimulation of the frontal cortex elicits hedonic vocalizations [21], whereas lesions reduce play behavior [22]. The antidepressant effect of fluoxetine on immobility in the TST was shown to correlate with BDNF expression in the frontal cortex, but not in the hippocampus [23] implicating a unique role for the frontal cortex in depression-like behavior of mice. Thus in the current study, expression of genes associated with inflammation were quantified in the frontal cortex of mice.

IGF-I is well recognized as a neuroprotective hormone and paracrine growth factor displaying activities in a variety of neuropathologies [24,25]. IGF-I is cleaved within the brain to release an N-terminal tripeptide [26]. GPE, like IGF-I, is neuroprotective both *in vivo *and *in vitro *[27]. Lower amounts of GPE provide protection when given i.c.v. compared to i.p. suggesting a central site of action; indeed GPE does not appear to act outside the nervous system. Thus GPE may represent a centrally active IGF-derivative, separating it from the global role of IGF-I. Importantly, purification of IGF-I from brain yields IGF-I lacking the first 3 amino acids [26,28,29] suggesting that GPE is naturally produced in the brain. Indeed, GPE is found in the normal brain [30] and a protease that releases GPE is found in brain [31,32]. Protease inhibitors, that prevent the release of GPE from IGF-I, block IGF-I inhibition of GNRH secretion [33,34]. This later finding strongly suggests that GPE mediates at least part of the central action of IGF-I. These finding suggest that GPE is a natural product of central IGF-I and has naturalistic neuroprotective actions. A central function for GPE, outside of cell survival such as behavior modification, has not been reported. The current study shows that like IGF-I its' natural cleaved product, GPE, has behavior modifying activity. In the present series of experiments, we show that both IGF-I and GPE administered centrally are able to prevent and cure depression-like behavior induced by peripheral administration of LPS.

## Methods

### Animals

Male CD-1 mice, 7 to 8 weeks old, were purchased from Charles River Laboratory International, Inc. (Wilmington, MA). Mice were group housed in ventilated cages under a 12:12 h reversed light:dark cycle (lights off at 10:00 h) for 2 weeks until surgery. Juvenile, 3 to 4 wk of age, C57BL/6J mice were used as novel targets for tests of social exploration. Food and water was provided *ad libitum*. Animal care and procedures were conducted with the approval of the University of Illinois' Institutional Animal Care and Use Committee.

### Surgery

Mice were anesthetized with ketamine (100 mg/kg body weight) and xylazine (10 mg/kg body weight). Pain was attenuated using buprenorphine (0.05 mg/kg body weight) given prior to surgery. Mice were secured in a stereotaxic instrument (David Kopf Instruments, Tujunga, CA). Stainless-steel guide cannulae (26-gauge, Plastics One Inc., Roanoke, VA) were implanted above the lateral ventricle; 0.6 mm posterior and 1.3 mm lateral to the bregma. The guide cannulae extended 1.3 mm below the skull. Cannulae were secured with "cold cure" Teets denture mixture (Co-oral-lte Dental MFG Co, Diamond springs, CA). Mice were individually housed post-surgery in conventional cages with a 2-week recovery period before treatment.

### Treatments

Mice were handled for at least 5 days prior to transfer to a room in the behavioral suite 2-3 days before treatment. A white noise Marpac soundscreen was used to minimize interference from external sounds while in the behavior suite. Experimental mice were 11 to 12 weeks of age at treatment. All treatments were administered at the end of light phase. IGF-I (recombinant human, GroPep, Adelaide, Australia) was prepared at 1 μg/μl, GPE was prepared at 5 μg/μl and LPS (serotype 0127:B8, Sigma, St. Louis, MO) at 33 or 83 μg/ml. IGF-I, GPE or 1 μl PBS were administered, injection time approximately 1 min, through the guide cannula and into the lateral cerebral ventricle using a 33-gauge stainless-steel cannula. Therefore, the final dose of IGF-I was 1 μg, which we have previously shown to be active i.c.v. [11], and the final dose of GPE was 5 μg, which is within the range tested i.c.v. for neuroprotection [35,36]. LPS was administered i.p. at 10 ml/kg body weight for final doses of 330 or 830 μg/kg body weight. We have previously shown that the higher dose of LPS causes depression-like behavior for at least 24 h assessed using either the forced swim, tail suspension or sucrose preference tests, while locomotor activity is expected to return to normal [10]. The low dose of LPS was determined empirically as a dose that elicits depressive-like behavior after a short acute-phase sickness response. At 330 μg/kg LPS body weight, mice do not present with either sickness or depression-like behaviors at 24 h (data not shown). IGF-I, GPE or PBS were administered either 30 min prior to 330 μg/kg LPS or PBS or they were administered 24 h after 830 μg/kg LPS. Treatment combinations are: PBS/PBS (Control), IGF-I or GPE/PBS (IGF-I or GPE), PBS/LPS (LPS) and IGF-I or GPE/LPS (IGF-I + LPS or GPE + LPS). LPS, at 830 μg/kg body weight, induces depression-like behavior that lasts at least 30 h. This time interval presents ample opportunity for IGF-I/GPE administration to be given post- LPS (at 24 h) while still permitting assessment of depression-like behavior. LPS, at 330 μg/kg body weight induces a less intense and more transient sickness response permitting the evaluation of an IGF-I/GPE effect on the development of depression-like behavior.

### Sickness response

Body weight and food weight were recorded before treatment and at various intervals post treatment. Sickness associated with LPS administration parallels a loss of body weight and decreased food intake. Social exploration of novel C57BL/6J juvenile mice and general locomotor activity were used to assess sickness behavior. Social investigation was performed with protected juveniles; juveniles confined to a 8 × 8 × 11.5 cm wire cage and placed in the corner of the experimental mouse's home cage [11]. Time spent by the experimental mouse exploring the caged juvenile during the 5 min test interval was recorded by a trained person blind to treatment. Social exploration was performed 24 h prior to treatment (baseline), then 2 and 6 h after treatment, using a different juvenile at each time point. Social exploration was used to assess recovery rate during the acute phase of sickness; 2-6 h after LPS administration because it can be administered repeatedly without habituation by the test subject. Locomotor activity (LMA) was recorded by a trained person blind to treatment from 5 min recording of mice placed in a clean cage and was only performed once for each experimental mouse 27 h after treatment with LPS. We use this test to determine the degree of residual sickness behavior when testing for depression-like behavior at a single time point usually 24 to 30 h following LPS. This test was used *in lieu *of social exploration because of its simplicity. LMA cannot be used to assess rate of recovery during the acute phase of sickness as it can only be performed once per mouse, due to habituation. LMA was assessed as the number of cage quadrants entered during the test period. All behavioral assessments were performed under red light illumination during the dark phase of the light cycle.

### Depression-like behavior

Depression-like behavior was measured as duration of immobility in the FST or TST and preference for consumption of a sweetened solution (sucrose preference). The TST was performed as previously described [11] using the Mouse Tail Suspension Package (MED-TSS-MS; Med Associates, St Albans, VT) 9 h after treatment with LPS. Adhesive tape was attached to each mouse's tail for suspension from a strain gauge. Force from the mouse's struggle was recorded during a 10 min session. Mice were considered immobile when force was below the lower threshold. The FST was performed as previously described [13] 30 h after treatment with LPS. Mice were recorded for 6 min and immobility recorded by a trained person blind to treatment. Sucrose preference was performed by quantifying disappearance of liquid (change in weight) from bottles containing either water or water containing 1% sucrose (wt./vol.). Preference was calculated as disappearance of the sucrose solution/disappearance of total fluid (water + sucrose). Four hour sucrose preference was measured from 12 noon to 4 pm. Animals were trained for 3 days prior to treatment; then preference was assessed on the same day as LPS treatment (4 h post-treatment) and the following day (4 h post-treatment with GPE).

### Tissue preparation for real time PCR

Immediately after assessment of behavior, mice were euthanized with CO_2 _then they were transcardially perfused with cold PBS. Brains were excised, dissected and frozen on dry ice. The frontal cortex, containing the frontal association, dorsolateral orbital, ventral orbital and prelimbic cortices (first 1 mm of the frontal cortex as defined in *The Mouse Brain *[37]), was collected and stored at -80°C until solubilized with TRIzol (Invitrogen Life Technologies, Carlsbad, CA) and RNA prepared as described [11]. RNA was quantified using a Nanodrop ND-1000 spectrophotometer (Nanodrop Technologies, Inc. Wilmington, DE). RNA was reverse transcribed using High Capacity cDNA Reverse Transcription Kits (catalog no. 4368813, Applied Biosystems, Foster city, CA) to prepare cDNA. Real-time rtPCR was performed to quantify steady-state mRNA as described previously [11]. Assays were purchased from Applied Biosystems (Foster City, CA) and amplification performed with a Prism 7900 (Applied Biosystems) and the TaqMan universal PCR master mix (Applied Biosystems, catalog no. 4305719). A 50 ng aliquot of cDNA was used per reaction. GAPDH was used to normalize target gene expression. Expression of GAPDH was not affected by any of the treatment combinations (data not shown). Changes in gene expression are expressed as 2^-ΔΔCts^, where Ct is the cycle threshold. A brief description of the target genes and primers has been presented [11].

### Statistical analysis

All data were expressed as mean ± SEM; Data were analyzed by a two-way (IGF-I × LPS) ANOVA using StatView (SAS Institute Inc., San Francisco, CA) or SigmaPlot (Systat Software, Inc., San Jose, CA). Where appropriate (social investigation, changes in body weight and food intake), data were analyzed using repeated measures ANOVA. SigmaPlot was used for the preparation of figures. Tukey's HSD (Honestly Significant Difference) was used for *post hoc *analysis when a significant interaction was present.

## Results

### IGF-I does not prevent LPS-induced sickness, but attenuates depression-like behavior

**Figure 1:**
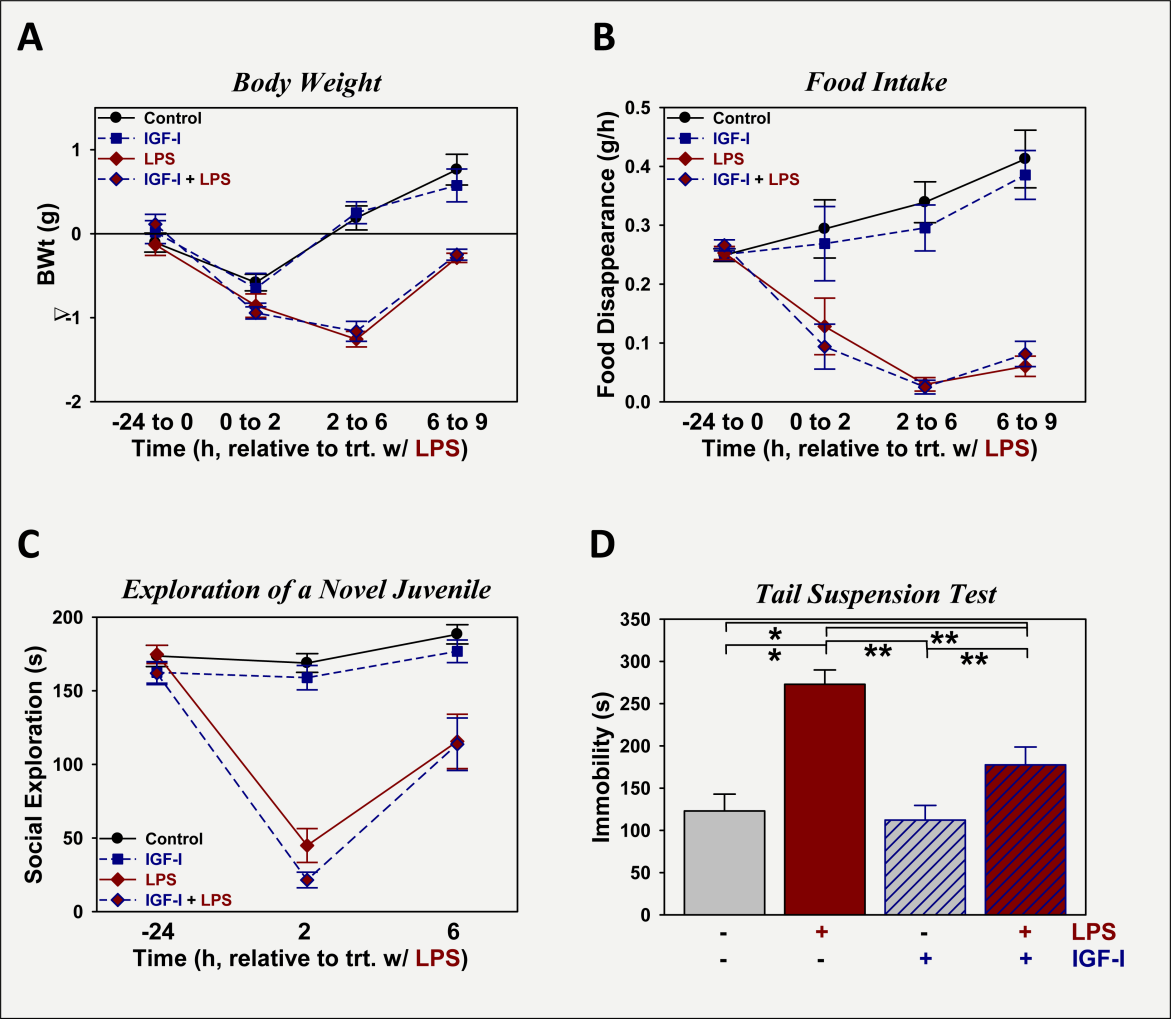
**IGF-I does not affect sickness, but prevents the development of depression-like behavior induced by LPS**. Mice were treated with vehicle or IGF-I (1 μg i.c.v.) followed 30 min later by vehicle or LPS (330 μg/kg body weight i.p.). Sickness was assessed as changes in body weight (A) and food intake (B), while sickness behavior was assessed as change in time spent investigating a novel juvenile (C). Depression-like behavior was quantified as the time of immobility during the FST, 9 h after treatment with LPS (D). n = 17-21 per treatment.

To assess its ability to prevent LPS-induced depression-like behavior, IGF-I was administered i.c.v. 30 min prior to LPS and several assessments were made to quantify sickness and depression-like responses. To investigate the effect of i.c.v. IGF-I on sickness, changes in body weight, food intake and social exploration towards a novel juvenile were assessed (Figure 1). As expected, LPS administration at 330 μg/kg body weight i.p. caused sickness, p < 0.05 evident by all measures. Mice treated with LPS lost body weight (Figure 1A), ate less food (Figure 1B) and spent less time exploring a novel juvenile mouse (Figure 1C). Mice were recovering between 6 and 9 h post-LPS for all three indices, although less-so for food intake. These are typical responses for this low dose of LPS. Administration of IGF-I i.c.v. did not alter the ability of LPS to induce sickness, being without effect on body weight, food intake and social explorations, p > 0.05. Depression-like behavior was assessed by duration of immobility during the tail suspension test (Figure 1D). Duration of immobility was increased by LPS (p < 0.05) and decreased by IGF-I (p < 0.05). The interaction between the two treatments was significant [F (1,80) = 4.9, p < 0.05]. *Post-hoc *analysis revealed that duration of immobility did not differ between controls and mice treated with IGF-I, although time spent immobile by mice treated with IGF-I + LPS was still above that of controls or IGF-I, p < 0.05; thus IGF-I did not completely block LPS-induced depression-like behavior. Duration of immobility was highest in mice treated with LPS and was significantly different from that of all other groups, p < 0.05. Thus, similar to our previous work administering both IGF-I and LPS i.c.v. [11], i.c.v. IGF-I has anti-depression like activity, but not anti-sickness activity against i.p. administered LPS.

### IGF-I does not affect preexisting LPS-induced sickness, but alleviates LPS-induced depression-like behavior

**Figure 2:**
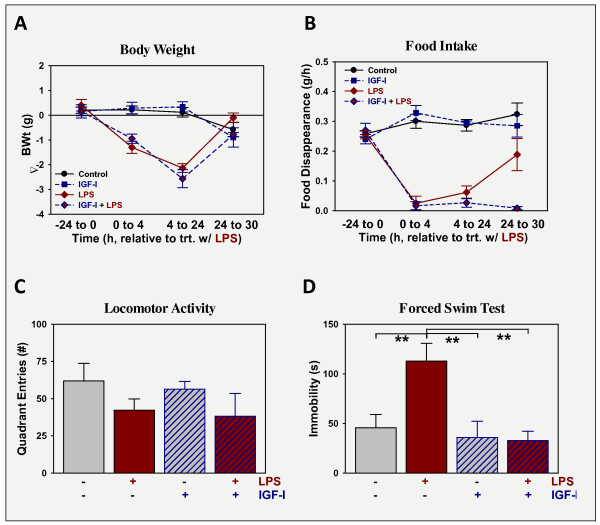
**IGF-I does not affect sickness, but relieves depression-like behavior induced by LPS**. Mice were treated with vehicle or LPS (830 μg/kg body weight i.p.) followed 24 h later by vehicle or IGF-I (1 μg i.c.v.). Sickness was assessed as changes in body weight (A) and food intake (B) while sickness behavior was assessed as change in general activity, 27 h after treatment with LPS (C). Depression-like behavior was quantified as the time of immobility during the FST, 30 h after treatment with LPS (D). n = 6.

To assess its ability to cure LPS-induced depression-like behavior, IGF-I was administered i.c.v. 24 h after i.p. LPS (when sickness behavior was expected to be mild or absent; thereby testing for an antidepressant activity of IGF-I following acute sickness). As in the prevention model, several assessments were made to quantify sickness and depression-like responses. As expected, LPS administration at 830 μg/kg body weight i.p. caused sickness, p < 0.05 evident by all measures. Mice treated with LPS lost body weight (Figure 2A), ate less food (Figure 2B). At 27 h, LPS-treated mice still had residual albeit mild sickness evident as lower spontaneous movement in a clean cage compared to controls: locomotor activity (Figure 2C). These are typical responses for this higher dose of LPS. Administration of IGF-I i.c.v. did not alleviate the changes induced by LPS during the 6 h time interval (24-30 h); IGF-I being without effect on body weight, food intake and locomotor activity, p > 0.05. Depression-like behavior was assessed by duration of immobility during the forced swim test (Figure 2D). Duration of immobility was elevated by LPS and this elevation in depression-like behavior was reversed by IGF-I with a significant interaction between the two treatments [F (1,21) = 5.5, p < 0.05]. *Post-hoc *analysis revealed that immobility did not differ between controls, mice treated with IGF-I, or mice treated with both IGF-I + LPS, p > 0.05. In contrast, immobility was highest in mice treated with LPS and was significantly different from all other groups, p < 0.05. Again, IGF-I had anti-depression, but not anti-sickness activity.

### GPE does not prevent LPS-induced sickness, but attenuates depression-like behavior

**Figure 3:**
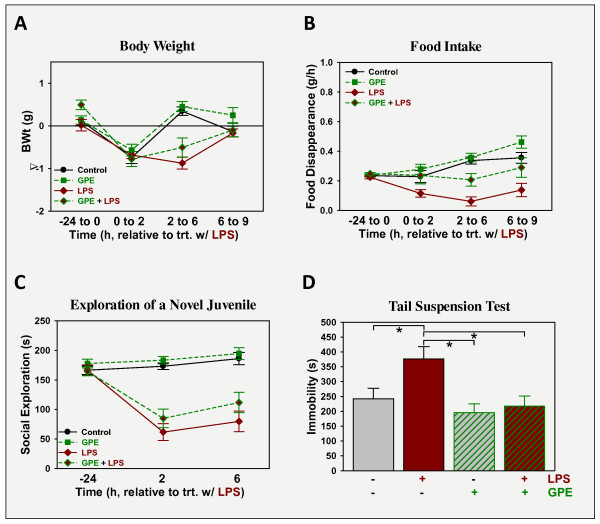
**GPE does not affect sickness, but prevents the development of depression-like behavior induced by LPS**. Mice were treated with vehicle or GPE (5 μg i.c.v.) followed 30 min later by vehicle or LPS (330 μg/kg body weight i.p.). Sickness was assessed as changes in body weight (A) and food intake (B), while sickness behavior was assessed as change in time spent investigating a novel juvenile (C). Depression-like behavior was quantified as the time of immobility during the FST, 9 h after treatment with LPS (D). n = 7-8.

To assess its ability to prevent LPS-induced depression-like behavior, GPE was administered i.c.v. 30 min prior to LPS to quantify sickness and depression-like responses. LPS administration i.p. caused sickness again evident by all measures, p < 0.05: mice treated with LPS lost body weight (Figure 3A), ate less food (Figure 3B) and spent less time exploring a novel juvenile mouse (Figure 3C). Mice recovered between 6 and 9 h post-LPS for all three indices. Administration of GPE i.c.v. did not alter the ability of LPS to induce sickness, being without effect on body weight, food intake and social explorations, p > 0.05. Depression-like behavior was assessed by duration of immobility during the tail suspension test (Figure 3D). Immobility was elevated by LPS (p < 0.05) and reduced by GPE (p < 0.05) although the interaction between the two treatments did not reach significance. These changes show that GPE has anti-depression like activity, but not anti-sickness activity.

### GPE does not affect preexisting LPS-induced sickness, but alleviates LPS-induced depression-like behavior

**Figure 4:**
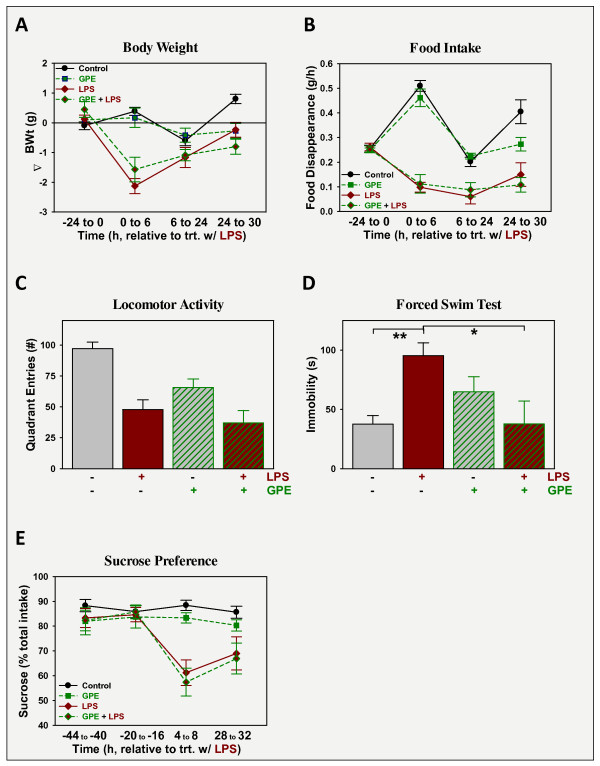
**GPE does not affect sickness, but relieves depression-like behavior induced by LPS**. Mice were treated with vehicle or LPS (830 μg/kg body weight i.p.) followed 24 h later by vehicle or GPE (5 μg i.c.v.). Sickness was assessed as changes in body weight (A) and food intake (B) while sickness behavior was assessed as change in general activity, 27 h after treatment with LPS (C). Depression-like behavior was quantified as the time of immobility during the FST, 30 h after treatment with LPS, or sucrose preference (D). n = 5-6.

To assess its ability to cure LPS-induced depression-like behavior, GPE was administered i.c.v. 24 h after i.p. LPS. As described above, LPS administration at 830 μg/kg body weight i.p. caused sickness, p < 0.05 evident by all measures: mice treated with LPS lost body weight (Figure 4A), ate less food (Figure 4B) and had lower spontaneous movement in a novel clean cage: locomotor activity (Figure 4C). Administration of GPE i.c.v. did not alleviate the changes induced by LPS; GPE being without effect on body weight or food intake during the 6 h time interval (24-30 h). GPE decreased locomotor activity, p < 0.05; without a significant LPS × GPE interaction, p > 0.05. Depression-like behavior was assessed by duration of immobility during the FST (Figure 4D). Duration of immobility was elevated by LPS. GPE had no effect on its own, on the duration of immobility during the FST, but there was a significant interaction between the effects of LPS and GPE [F (1,19) = 8.5, p < 0.05]. *Post-hoc *analysis revealed that immobility did not differ between controls, mice treated with GPE, or mice treated with both GPE + LPS, p > 0.05. In contrast, duration of immobility was highest in mice treated with LPS and significantly different from controls or mice treated with GPE plus LPS, p < 0.05. Again, GPE had anti-depression activity. In contrast to these findings, GPE was unable to reverse the decrease in sucrose preference observed following LPS administration. As shown in Figure 4E, sucrose preference was stable for the two days prior to treatment for all treatment groups. LPS caused a decrease in sucrose preference during the 4 to 8 h interval after treatment. Sucrose preference was still depressed for LPS-treated mice when assessed 28 to 32 h after treatment. GPE, given 24 h after LPS, did not alter sucrose preference 4 to 8 h later (28 to 32 h post-LPS).

### GPE administered i.c.v. decreases i.p. LPS-induced expression of brain cytokines

**Figure 5:**
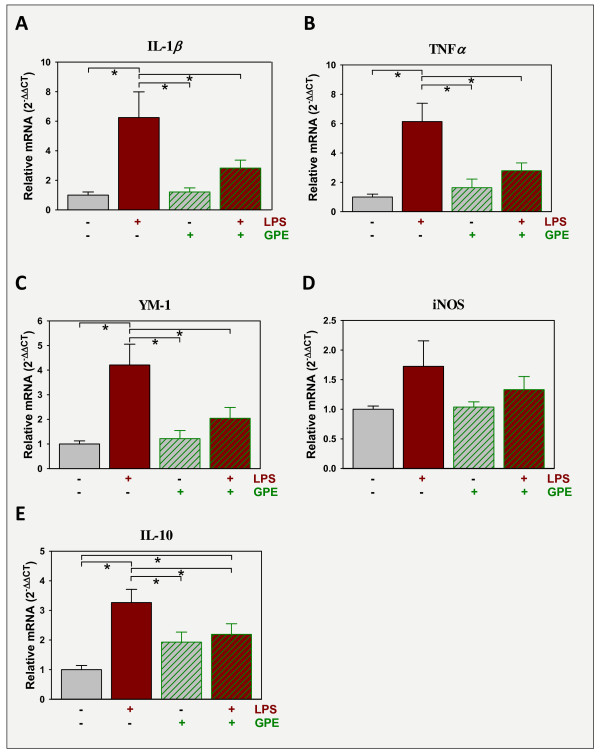
**GPE blocks the expression of inflammatory markers that are induced by LPS**. Mice were treated with vehicle or GPE (5 μg i.c.v.) followed 30 min later by vehicle or LPS (330 μg/kg body weight i.p.). Mice were sacrificed, perfused and brains collected 9 h later. Steady-state mRNA expression of pro-inflammatory markers, IL-1β (A), TNFα (B), YM-1 (C) and iNOS (D), was quantified by real-time rtPCR in the prefrontal cortex and expressed relative to GAPDH. GAPDH expression was constant across treatment, p > 0.5, and changes in gene expression shown in Figures 5, 6, and 7 reflect treatment-induced effects on target gene expression. n = 11-12.

LPS-induced changes in behavior are dependent on the induction of brain pro-inflammatory cytokines. We had previously shown that IGF-I diminishes expression of brain cytokines [11]; a possible mechanism to alleviate depression-like activity. Thus the ability of GPE to alter the expression of inflammatory factors within the frontal cortex was examined. LPS (Figure 5) significantly (p < 0.05) increased mRNA expression of IL-1β and TNFα (Figure 5A and 5B, respectively). In both cases, GPE alone did not affect basal cytokine expression (p > 0.05), but significantly attenuated the LPS induction of TNFα (interaction between LPS and GPE, [F (2,42) = 7.2, p < 0.05]) and approached significance for IL-1 β (interaction between LPS and GPE [F (2,42) = 3.7, p = 0.06]). Expression of YM-1, a marker of microglia activation [38], was also elevated by LPS (Figure 5C). Similar to TNFα, there was an interaction between LPS and GPE for YM-1 expression [F (2,42) = 5.5, p < 0.05]. *Post-hoc *analysis indicated that GPE attenuated LPS induction of YM-1. LPS increased iNOS expression (p < 0.05). Although GPE appeared to reduce expression of iNOS, the effect of GPE and its interaction with LPS was not significant (Figure 5D). IL-10 expression was induced by LPS and attenuated by GPE with a significant interaction between LPS and GPE [F (2,42) = 8.6, p < 0.05]. *Post-hoc *analysis indicated that GPE did not return the level of IL-10 expression to that of controls, but there was no significant difference between expression in mice treated with GPE or LPS + GPE (Figure 5E) Overall, these data indicate that glial activation is suppressed by central GPE, offsetting the inflammatory response initiated by i.p. LPS.

### GPE administration does not regulate expression of tryptophan dioxygenases

**Figure 6:**
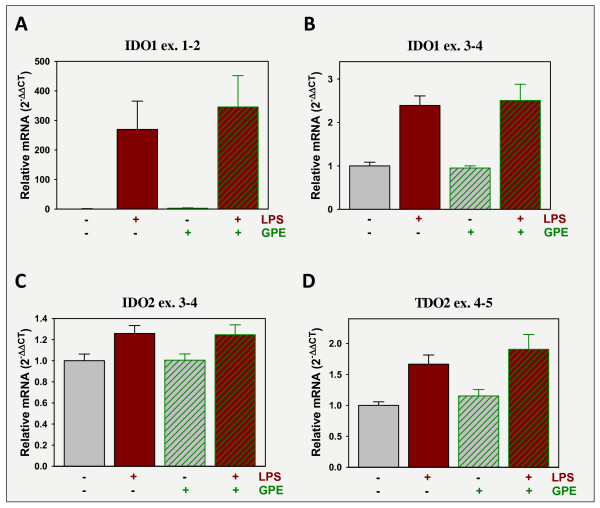
**LPS increases the expression of tryptophan dioxygenases, but GPE does not attenuate this response**. Mice were treated as in Figure 5. Steady-state mRNA expression, of three enzymes that metabolize tryptophan to kynurenine, IDO1 (A, B), IDO2 (C) and TDO2 (D), was quantified by real-time rtPCR in the prefrontal cortex and expressed relative to GAPDH. n = 11-12.

To assess the possible involvement of changes in tryptophan metabolism in LPS-induced depression-like behavior and GPE's antagonistic actions, expression of IDO1, IDO2 and TDO2 were quantified in the frontal cortex. Assays designed to probe for expression of IDO1's full-size transcript (probe spanning exons 1 and 2) and one that quantified all transcripts (probe spanning exons 3 and 4) used to quantify IDO1 expression. Expression of IDO1 ex. 1-2 was increased almost 300-fold by LPS, p < 0.05, but unaffected by GPE (Figure 6A). Expression IDO1 ex. 3-4 amplification was increased by LPS to ~2.5-fold of controls, p < 0.05, but expression again was unaffected by GPE (Figure 6B). IDO2 and TDO2 expression were quantified with probes that span exons 3 and 4 and 4 to 5, respectively. These assays quantify all transcripts. Expression of IDO2 (Figure 6C) and TDO2 (Figure 6D) were both increased by LPS, p < 0.05, but unaffected by GPE. These data indicate that GPE is not altering depression-like behavior by reducing the LPS-induction of tryptophan dioxygenase mRNA expression.

### GPE administration does not regulate expression of either IGF-I or BDNF

**Figure 7:**
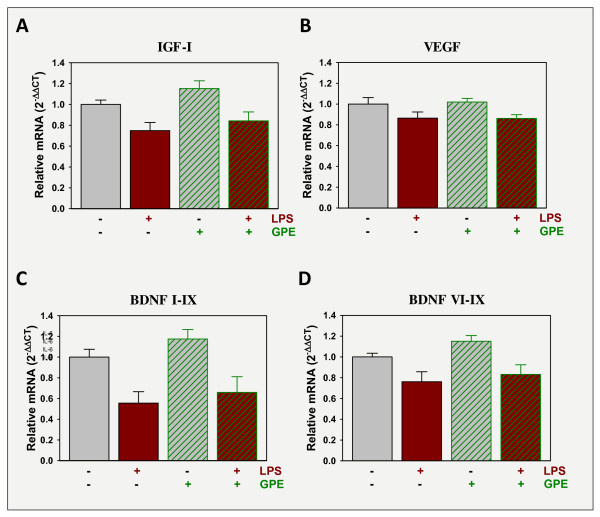
**LPS depresses the expression of neurotrophic factors, but GPE does not alter the LPS response**. Mice were treated as in Figure 5. Steady-state mRNA expression of several neurotrophic factors, IGF-I (A), VEGF (B) and BDNF (C, D), was quantified by real-time rtPCR in the prefrontal cortex and expressed relative to GAPDH. n = 11-12.

To assess the possible involvement of changes in neurotrophin expression in the GPE behavior response, expression of IGF-I, VEGF and BDNF were quantified. IGF-IEb was decreased by LPS, p < 0.05, but unaffected by GPE (Figure 7A). VEGF was also decreased by LPS, p < 0.05, but again GPE had not effect (Figure 7B). BDNF is synthesized from multiple transcripts within the brain. LPS decreased the expression of both of the transcripts that were measured, BDNF exon I-IX (Figure 7C) and BDNF exon VI-IX (Figure 7D). Neither mRNA transcript for BDNF was affected by GPE. Thus, although LPS clearly decreases neurotrophin expression, GPE does not reverse this action.

## Discussion

Three new findings are evident from the current studies, 1) although they both share an inflammatory component, sickness and depression-like behavior are independently modulated by the IGF system, 2) when added exogenously, two naturally occurring products found in the naïve brain, mature IGF-I and GPE, act centrally to temper LPS-induced changes in depression-like behavior, and 3) GPE can temper the central innate immune response to LPS providing a possible mechanism by which to regulate depression-like behavior. A direct behavior modifying role of GPE has never been reported. Previous studies with the GPE peptide had shown biological activity associated with neuroprotection using preclinical models for Alzheimer's, Parkinson's disease, stroke, multiple sclerosis and Huntington's [27], but a central behavioral effect that is independent of neuroprotection had yet to be reported. Neither IGF-I or GPE affected behavior of naïve (non-LPS) mice, but had anti-depressant activity when tested against the inflammation-inducer LPS in the TST and FST. It's not known whether the effect of GPE can be extended to other models of depression as GPE was not able to reverse LPS-induced decrease in sucrose preference, used as an index of anhedonia.

Other studies have shown that IGF-I has anti-depressant activity using various rodents models. Voluntary exercise increases blood and brain IGF-I levels. This increase is in part responsible for exercise-induced anti-depressant activity [39]. An anti-IGF-I antibody blocked the decreased time of immobility during the FST of exercised mice [40], indicating that exercise-induced increases in IGF-I has a naturalistic and endogenous role in controlling behavior. Similarly, rough-and-tumble play of rats increases IGF-I levels in the frontal and parietal cortex and also increases hedonic 50-kHz ultrasonic vocalizations [41]. The play-induced increase in hedonic vocalizations was dependent on IGF-1R activation and i.c.v. administration of IGF-I increased hedonic vocalizations and decreased approach latency to self-administered play. IGF-I also decreased time of immobility in the FST, decreased consumption latencies in the novelty-induced hypophagia test using naïve mice and increased sucrose consumption of mice subjected to chronic unpredictable stress [40,42-44]. Activating endogenous IGF-I, by freeing it from inhibitory IGF-binding proteins with NBI-31772, decreased immobility in the TST. The action of IGF-I and NBI-31772 was blocked by JB1, an IGF-1R antagonist [44] supporting a mechanism that requires activation of IGF-1R. A single i.c.v. injection of IGF-I to rats decreased immobility during the FST [42] while JB1 blocked the anti-depressant activity of IGF-I [43]. Data such as these, especially with naïve animals, show that the behavior modifying role of endogenous and exogenous IGF-I is independent of IGF-I's well documented neuroprotective actions and that the anti-depressant activity of IGF-I can be quantified with several tests that have proven to be reliable indices of depression-like behaviors. Blocking the anti-depressant activity of exogenous IGF-I with JB1, which prevents IGF-1R receptor activation, shows that IGF-1R activation is requisite for IGF-I action, but does not show whether all the effects are mediated by the IGF-1R. With this in mind we determined whether the naturalistic product of IGF-I cleavage within the brain that acts independent of the IGF-1R [45] can exert additional anti-depressant activity.

In the current study, GPE had very similar actions compared to IGF-I. GPE, given before or after LPS, elicited an anti-depressant effect with mice using either the TST or FST. Like IGF-I, GPE did not alter the sickness response to i.p. LPS. The lack of an effect of these peptides on sickness is not unexpected. LPS given i.p. elicits a strong peripheral immune response and also sends inflammatory-initiating signals to the brain via the vagus nerves and other immune-to-brain communication pathways [46]. The acute peripheral immune response, subsequent delivery of cytokines to the brain via the humoral and neural communication pathways is unlikely to be altered by i.c.v. administration of either IGF-I or GPE. Thus in this model, the sickness response appears to be largely under the control of the peripheral inflammatory input to the brain. These primary signals subsequently initiate an activation of the innate immune system of the brain which is tempered by GPE (Figure 5). Decreased neuroinflammation in response to IGF-I [11] and GPE (current study) correlate with changes in depression-like behavior when assessed using the TST and FST. The anti-depressant activities of IGF-I and GPE in the current experiments assessed with the TST and FST does not appear to be a result of an overall attenuation of psychomotor retardation as locomotor activity was unaffected by either IGF-I or GPE. GPE was unable to alter mouse performance in a test for anhedonia. Sucrose preference was not affected by GPE. Attempts to assess the activity of IGF-I on sucrose preference were complicated by the ability of an acute IGF-I challenge to enhance sucrose consumption in naïve mice (data not shown), reflecting its insulin-like activity and ability to directly alter glucose metabolism. Differential effects with the TST and FST versus sucrose preference suggest that GPE plays a specific role in alleviating only some symptoms of inflammation-induced depression, helplessness/despair, without affecting another, anhedonia. An hedonic response may require mature IGF-I or chronic exposure. In a study by Duman, chronic subcutaneous infusion of IGF-I (~1-1.5 mg/day for 14 d, vs. a 1 mg bolus in the current work) did not increase sucrose consumption in naïve mice (an effect we have found with bolus IGF-I injection i.c.v.), but this constant chronic infusion of IGF-I reversed the lowered sucrose consumption by mice subjected to chronic unpredictable stress [40]. Our new finding however provides a novel model to help define central pathways controlling specific behavioral symptoms.

We previously found that IGF-I induced the expression of several BDNF transcripts [11]; another possible mediator of its anti-depressant activity. One of the more interesting aspects of BDNF biology is the differential expression and regulation of specific transcripts. All transcripts produce mature BDNF, but in different cells types. Transcripts initiating from exons I, II and III are expressed predominantly in neurons and transcripts initiating from exons IV, V and VI are expressed by both neurons and astrocytes [47]. Normal expression of all transcripts may be necessary for wellness, as a knockout of even a single transcripts causes depression-like behavior of mice [48]. Duloxetine, an SNRI class anti-depressant, increases the expression of only 4 of the 9 transcripts [49] indicating specificity of treatment. Thus we again examined multiple BDNF transcripts. However, GPE did not induce BDNF expression and thus BDNF may not play a significant role in the anti-depressant role of GPE. Only a single dose of GPE was tested in the present study; a dose with proven neuroprotective action [35,36]. The present results call for a more extensive investigation of the dose-response effect of GPE on inflammation-induced depression and moreover on the ability of endogenously generated GPE to mediate part of the anti-depressant activity of IGF-I.

Clearly from studies mentioned above, the anti-depressant activity of mature IGF-I is dependent of IGF-1R signaling, which also mediates IGF-I's neuroprotective action. The receptor that mediates GPE action on behavior has not been characterized. GPE was shown to displace glutamate from its receptor, but GPE's neuroprotective activity is not clearly related to glutamate/NMDA receptor (NMDA-R) binding as it competes with glutamate binding with a very low affinity (30 μM) even though neuroprotective activity is evident at 0.1 nM [33,34,45,50,51]. This discrepancy suggests that GPE activity is dependent on an as yet uncharacterized mechanism [52]. Peptidase inhibitors that block processing of many proteins including the release of GPE from intact mature IGF-I with the generation of des-(1-3)-IGF-I, block IGF-I inhibition of GNRH secretion [33,34] suggesting that GPE mediates at least part of the central action of IGF-I. Mature intact IGF-I has antidepressant activity, but it is still not known if des-(1-3)-IGF-I, acting via the IGF-1R, or the generation of GPE from exogenous IGF-I play a significant role in the antidepressant activity of the intact mature peptide. However, since blockage of IGF-1R activity abrogates anti-depressant activity of the mature peptide [44] and GPE does not activate the IGF-1R [45], clearly IGF-I itself has anti-depressant activity through the IGF-1R independent of GPE. Thus it appears that IGF-I has the potential to generate two distinct signaling events that result in anti-depressant activity; an IGF-1R mediated action and an IGF-1R independent GPE-mediated action.

One common link between IGF-I and GPE that may be related to their anti-depressant activity is their common ability to attenuate the activity of quinolinic acid [53,54]. Induction of tryptophan dioxygenase activity by inflammation plays a major role in depression as shunting of tryptophan metabolism toward the generation of metabolites such as quinolinic acid has been linked to inflammation-associated depression [55]. Using mice as a preclinical model, i.p. LPS induces IDO1 activity in the periphery and brain [56] with an increase in central and plasma kynurenine/tryptophan ratios [13]. Increased IDO1 expression is associated with the generation of kynurenine metabolites (kynurenic acid, 3-hydroxykynurenine and quinolinic acid) that are active neuromodulators. Increased levels of these modulators are associated with inflammation-dependent depression [57]. It is not clear as to which of these metabolites have a causative role in depression or if periphery or brain-generated tryptophan metabolites are responsible for depression-like behaviors but induction of peripheral or central IDO1 expression correlates with depression-like behavior of mice. IDO1 expression was quantified using two qPCR assays. IDO1 protein is translated from multiple transcripts with the full length transcript being less widely distributed in the body than other transcripts [58]. Although not reported for brain samples, in the placenta the major transcript does not include much of exon 1 [59]. An assay designed to probe for expression of IDO1's full-size transcript (probe spanning exons 1 and 2) resulted in weak amplification in controls (Ct = 38.7). Expression of all major IDO1 variants was quantified using a probe that spans exons 3 and 4. IDO1 ex. 3-4 amplification is much stronger in control brain samples, Ct = 27.4, than that of IDO1 ex. 1-2. IDO2 and TDO2 expression were quantified with probes that span exons 3 and 4 and 4 to 5, respectively. These assays quantify all major transcripts. Amplification of IDO2 in control mice was slightly stronger than that for IDO1 ex. 3-4 with a Ct value of 24.4, whereas amplification of TDO2 was weaker, Ct = 30.5. Although Ct values do not imply differences in mRNA levels within samples, because of probable differences in assay-to-assay qPCR efficiencies, the extremely low Ct value for IDO1 ex. 1-2 compared to all other dioxygenases indicate that in control animals it may be a minor source for protein translation. However, expression of IDO1 ex. 1-2 may play an important role in neurophysiology because of its strong induction with neuroinflammation; in this case following administration of LPS. In our current model, IDO1, IDO2 AND TDO2 mRNA expression, which are rate-limiting enzymes for the conversion of intracellular tryptophan to kynurenine, were all increased by LPS. Expressions were unaffected by GPE; similarly IGF-I did not attenuate LPS-induced dioxygenase expression (unpublished data). These data seem to suggest that IGF-I and GPE do not act by altering the consequences associated with elevated dioxygenase expression. However, it remains to be determined if IGF-I or GPE alter the effects of elevated tryptophan dioxygenase expression downstream of the enzyme; i.e. by directly attenuating the activity of quinolinic acid or other downstream products such as 3'-hydroxykynurenine or kynurenic acid.

Cytokine and neurotrophin mRNA expression was quantified in the prefrontal cortex as this region has been strongly implicated in the regulation of positive affect. In rodents, electrical stimulation of the frontal cortex elicits hedonic vocalizations by rodents, [21], whereas lesions reduce rough-and-tumble play behavior [22] both associated with a positive emotional state. Although many studies show a strong correlation between changes in the frontal cortex and hippocampus, the antidepressant effect of fluoxetine was shown to correlate with BDNF expression in the frontal cortex, but not in the hippocampus [23]. With humans, several positive affective stimuli increase neuronal activity in the frontal cortex [17-20]. Here we show that LPS induces expression of central cytokine and GPE reduces their expression in a brain region intimately associated with depression-like behaviors. The opposing effects of LPS and GPE on cytokine expression parallel changes in depression-like behavior.

The neurotrophic hypothesis for depression asserts that neuronal plasticity is dysfunctional because of insufficient neurotrophic support and that classic anti-depressants ultimately reactivate plasticity in the brain [60-62]. IGF-I is a classic neurotrophin; inducing the proliferation, survival, differentiation and maturation of cells of the central nervous system [63] and directly supporting neuronal plasticity [64]. GPE is also a neurotrophin; although characterized primarily by its ability to support neuronal survival and stimulation of glia proliferation [65]. IGF-I is expressed by neurons within the brain [66-68] and expression was reduced by the administration of LPS (Figure 7). This reduction would translate into low central levels of both IGF-I and GPE within the frontal cortex. This reduced protein expression would result in lower neurotrophic support along with the lower expression of BDNF and VEGF (Figure 7). By administration of IGF-I or GPE neurotrophic support is exogenously provided and depression-like behavior is attenuated.

In conclusion, data in this manuscript show that exogenous central administration of either IGF-I or GPE results in anti-depressant-like activity, assessed using the TST and FST. The behavioral changes did not occur with naïve mice but these neurotrophins attenuated the behavioral changes induced by i.p. LPS. These data extend the anti-depressant activity of the IGF system by showing that a proteolytic product derived from IGF-I, GPE, has anti-depressant activity paralleling that of the mature peptide. Both IGF-I and GPE offset the inflammatory response elicited by LPS which may in part be responsible for anti-depressant activity.

## Competing interests

RD has received honoraria from Bristol-Myers Squibb and Lundbeck Laboratories and works as a consultant for Lundbeck Laboratories. RD and KWK have received honoraria from Astra-Zeneca.

## Authors' contributions

RHM designed the experiments, performed surgery, treated animals, performed behavior tests, analyzed data and wrote much of the manuscript. SP performed surgery, treated animals, performed behavior tests, analyzed data and did all the PCR analyses. ML, KWK and RD helped in the design and interpretation of the experiments and editing the manuscript. All authors have read and approved the final version of this manuscript.
